# Influence of Unit Water Content Control on Concrete Performance in the Ready-Mixed Concrete Production Process

**DOI:** 10.3390/ma17040834

**Published:** 2024-02-09

**Authors:** Sang-Hoon Park, Hwa-Sung Ryu, Won-Jun Park

**Affiliations:** 1 Department of Safety & Disaster Management, Sungkyunkwan University, Suwon 16419, Republic of Korea; okpshppp@skku.edu; 2 Hanyang Experiment and Consulting, Hanyang University, Ansan 15588, Republic of Korea; 3 Department of Architect, Kangwon National University, Samcheok 25913, Republic of Korea

**Keywords:** ready-mixed concrete, unit water content, water addition, water–cement ratio, compressive strength, durability

## Abstract

This study examined the effects of increasing concrete unit water content and artificially controlling water content on concrete performance in the production process of ready-mixed concrete. Results showed that changes in the unit water content of 20 concrete mix proportions without air-entraining significantly reduced concrete compressive strength, increased porosity, and in-creased occurrence of bleeding. A unit water content increase of 25 kg/m^3^ or more may reduce the compressive strength of concrete below the design standard and significantly affect the occurrence of bleeding water. Moreover, an extra unit water content of at least 25 kg/m^3^ could significantly affect the diffusion of chloride ions in the concrete. The carbonation depth of concrete was extremely high with the increase in unit water content and water addition. In the production of concrete requiring at least normal strength or durability, the extra water change to total unit water content should be maintained at 15 kg/m^3^ or less. And a water–cement ratio of 48% or less and a unit water content of 155 kg/m^3^ or less are considered effective for management of concrete quality. Considering the aggregate type, absorption rate, and moisture state, the management of unit water content error in concrete production processes requires greater.

## 1. Introduction

Currently, ready-mixed concrete (hereafter referred to as concrete) is a vital construction material and standardized product used extensively in various applications and scales. The performance of concrete is dominantly influenced by the physicochemical properties and proportions (mixed) of its constituents [1]. Nataraja and Lelin [2] examined the performance of concrete by differentiating between fresh and hardened concretes [2]. Specifically, they reviewed the effects of constituent materials (such as binder, aggregate type, fiber reinforcement, and chemical admixtures) on the workability, rheology, and slump of fresh concrete and on the compressive strength and durability of hardened concrete. Among these, the amount of water and the water–cement ratio are closely related to concrete behavior. Hover [3] reported that the mechanisms of concrete deterioration and its resistance vary with the initial moisture content. The author mentioned the importance and effectiveness of water control and curing in concrete mixed design, considering the impact of water on concrete strength, shrinkage, and workability. Simultaneously, water is a crucial element in most mechanisms that degrade concrete properties [3,4,5].

Numerous empirical formulas have been developed to explain the relationship between concrete strength and water–cement ratio. Abrams [6] proposed a formula to estimate concrete strength based on water–cement ratio, and Popovics [7,8] proposed a new type of strength formula with independent variables such as cement content, moisture content, or paste content, thereby enhancing accuracy in strength estimation. Additionally, formulas have been proposed to demonstrate the impact of porosity, specifically air content, on concrete strength. Seitl et al. [9] discovered that the water-to-cement (W/C) ratio significantly influences the fatigue and mechanical properties of concrete. Marar and Özgür [10] analyzed the impact of the water–cement ratio centered around the unit cement amount in conditions without admixtures and confirmed its dominant influence on the slump, unit weight, and compressive strength of concrete. Further, studies on water content control in concrete mixtures, maintaining the unit cement amount constant, have focused on aggregates, their absorption rates, and unit water content. When using recycled aggregate, the moisture state of the aggregate and unit water content significantly reduce the concrete strength [11,12,13]. Aliabdo et al. [14] examined additional water contents in the range of 10–35 kg/m^3^ and reported a strength reduction of 20% or more.

Moreover, an increase in unit water content within concrete increases the occurrences of bleeding and drying shrinkage, ultimately affecting the durability of the concrete [3,4,5]. Consequently, various methods (such as high-frequency heating and microwave methods) have been proposed to quantitatively verify concrete unit water content [15,16,17,18]. In Korea, the standard for examining unit water content in concrete quality inspection is within ±20 kg/m^3^ [19]. In Japan, concrete quality is managed by regulating unit water content fluctuations to ±10, 15, and 20 kg/m^3^ [20]. The unit water content standards in BS (British Standard) and ACI (American Concrete Institute) are as follows [21,22,23,24]. In the BS method, water content is selected from the table specified in this standard for different consistency classes and different sizes of aggregates and types of aggregate: crushed or uncrushed. A reduction in the water content is also specified when additives are used with cement. In this method, when coarse and fine aggregates are of different types, the water content can be expressed as

W = (2W_f_/3) + (W_c_/3) (1)

where W_f_ = water content appropriate to type of fine aggregate, and W_c_ = water content appropriate to the type of coarse aggregate.

In the ACI method, rounded coarse aggregates generally require 18 kg less water for non-air-entrained and 15 kg less for air-entrained concretes. The use of water-reducing chemical admixtures may also reduce mixing water by 5% or higher.

This study investigated the control of concrete unit water content in the production process of ready-mixed concrete supplied to construction sites. It considered how the unit water content, as reported in numerous documents or regulations, is controlled during the manufacturing of ready-mixed concrete. We examined the effects of increased unit water content on concrete performance due to quality control errors (or mechanical errors) and artificial increases in unit water content. Specifically, we experimentally evaluated the impact of unit water content on the mechanical properties and durability of concrete without air-entraining (AE) agents.

## 2. Experimental Design

### 2.1. Concrete Mix Proportion

The basic mixed designs for the experiment are five types selected from a concrete manufacturing plant. These designs are recently supplied ready-mixed concrete mix proportions for construction projects. The designs have unit water contents in five stages (155, 165, 175, 185, and 195 kg/m^3^). Table 1 presents 20 mix proportions, including additional increases in unit water content (15, 25, and 35 kg/m^3^). In all mixes, the unit cement amount is fixed at 320 kg/m^3^, and the W/C ratio range is examined from 48.4 to 60.9. OPC type-Ⅰ cement was used, and aggregates were brought in and used from the concrete plant. Their properties are presented in Table 2. A twin-type mixer was used for concrete mixing, and the mixing time per batch was standardized for 3 min.

### 2.2. Experimental Items

**Figure 1:**
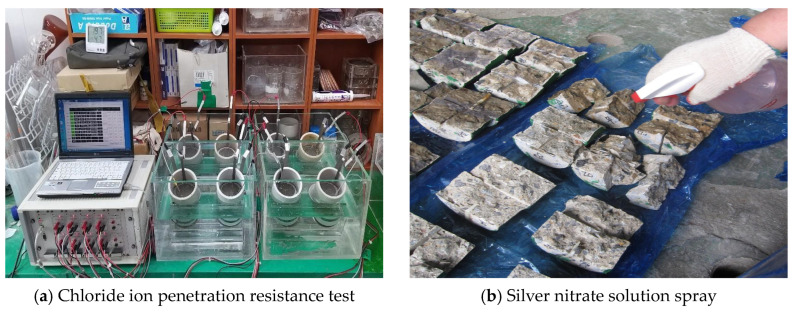
Measurement of chloride ion penetration depth.

The main concrete experimental items and evaluation criteria are presented in Table 3. For concrete mixes without AE agents, age-specific compressive strength, chloride ion penetration resistance, accelerated carbonation resistance, and porosity were evaluated. Compressive strength was measured following KS F 2405 [25], based on water curing periods (ages: 3 d, 7 d, 28 d). The chloride ion penetration resistance test followed NT BUILD 492 [26]. For the concrete specimens, approximately 50 mm from the surface was cut off, and the specified accelerated test was conducted, followed by measuring the depth of chloride ion penetration after the test. The chloride ion diffusion coefficient was calculated using the formula specified in NT BUILD 492. The penetration depth measurement process is shown in Figure 1. The accelerated carbonation test was conducted following KS F 2584 [27] and KS F 2596 [28]. Carbonation acceleration was conducted in a chamber with conditions of 40 °C temperature, 60% RH, and 5% CO_2_ concentration, and carbonation depth was measured at 7, 14, 21, and 28 d of age. The porosity measurement of concrete was performed using a mercury intrusion penetrometer. A sample of approximately 5 mm, excluding aggregates from the concrete, was immersed in iso-propylene for 24 h and then underwent a pre-treatment process of drying in a chamber at 60 ± 5 °C for 24 h to 48 h.

## 3. Results and Analysis

### 3.1. Compressive Strength

**Figure 2:**
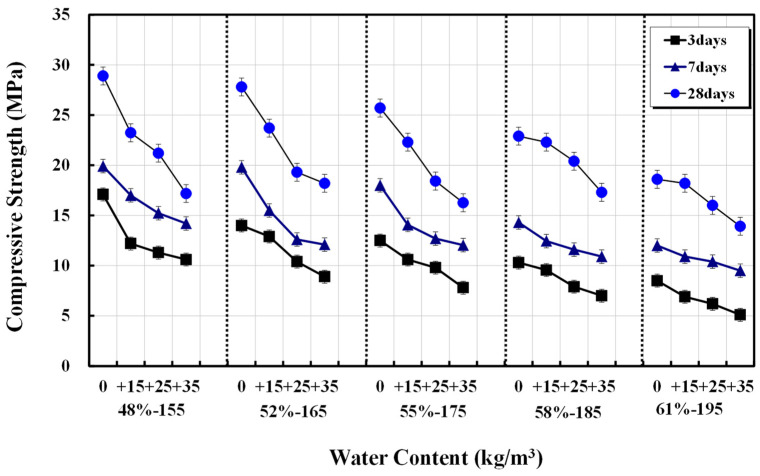
Increase in unit water content and concrete compressive strength.

**Figure 3:**
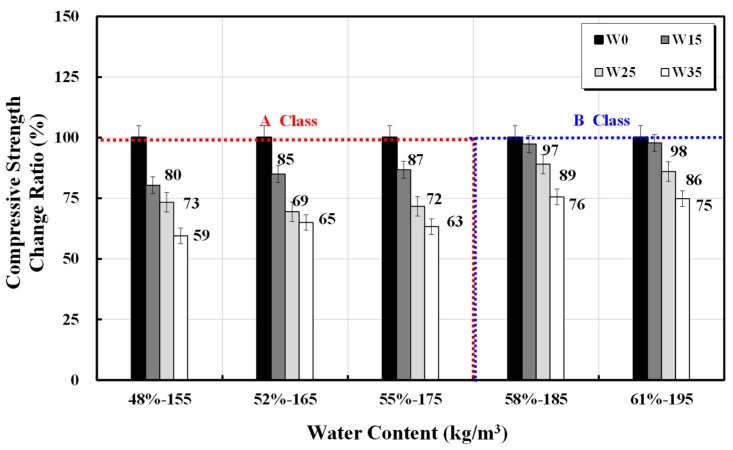
Change in concrete compressive strength with unit water content and water addition.

Figure 2 shows the age-specific compressive strength test results for the 20 mixed designs with 15, 25, and 35 kg/m^3^ increases in unit water content for the five types of basic mixed designs. The higher the additional unit water content, the greater the reduction in compressive strength. Figure 3 shows the relative compressive strength ratio compared with the 28 d compressive strength of the basic unit water content mix. The strength areas are divided into Classes A and B based on the basic unit water content range. For Class A, with a basic unit water content range of 155–175 kg/m^3^, the compressive strength decreased by 80–90%, 70–75%, and 60–65% with each addition of 15, 25, and 35 kg/m^3^ of unit water content, respectively. The calculation of concrete mixed strength considers the design standard strength and safety factor. Although this study focuses on concrete mixes without AE agents, an additional unit water content of 25 kg/m^3^ or more may reduce compressive strength below the design standard. A compressive strength reduction in the relatively lower strength area, Class B, was less than 5%, 10–15%, and 20–25%, but simultaneously, the occurrence of bleeding water was high. Thus, the strength reduction due to the additional unit water content was relatively low.

### 3.2. Chloride Ion Diffusion Coefficient

**Figure 4:**
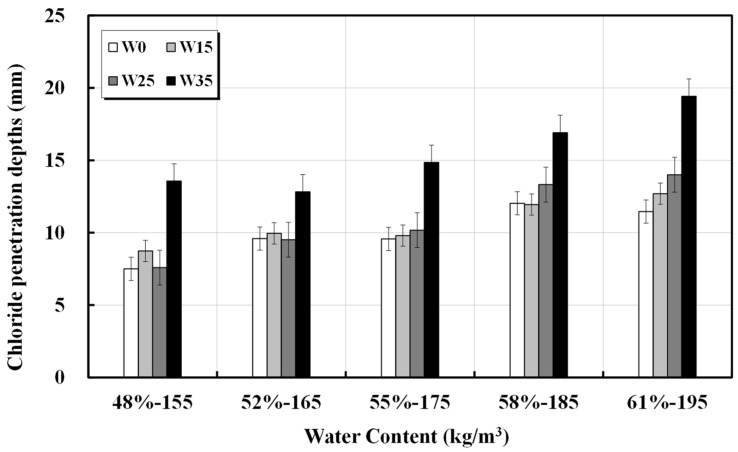
Depth of chloride ion penetration against unit water content.

**Figure 5:**
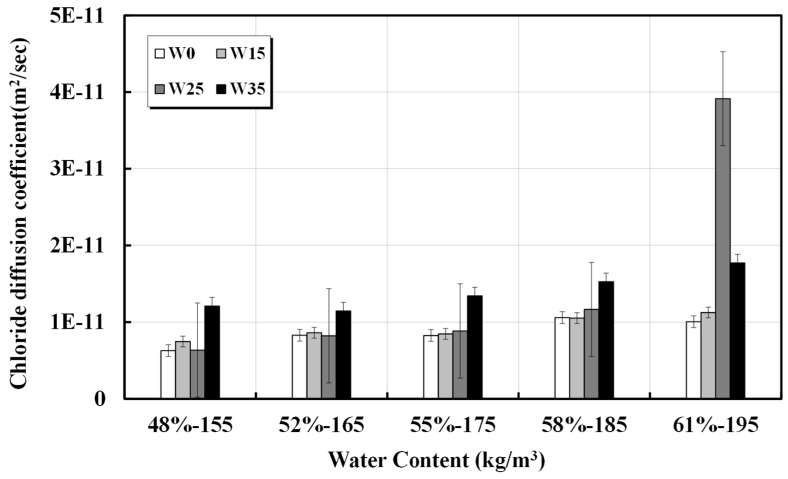
Diffusion coefficient of chloride ion according to unit water content.

The results of the chloride ion penetration resistance test conducted following NT BUILD 492 are presented in Table 4 and Figure 4. For concrete with a unit water content within the range of 155–175 kg/m^3^, the penetration depth and diffusion coefficient appeared to be similar to the additional unit water content, suggesting that structural durability can be ensured. However, concrete with a unit water content of 185 kg/m^3^ or higher exhibited a deeper chloride ion penetration depth. For concrete mixed designs requiring high durability, a unit water content of 175 kg/m^3^ or less is considered effective. Even if the water content inside the concrete increases because of artificial water addition or errors in the production process (factory), in the normal strength area, caution is required for an increase of at least 25 kg/m^3^ in unit water content. The calculated diffusion coefficients are presented in Table 5. The results calculated using the diffusion equation considering time dependence are shown in Figure 5 [30].

### 3.3. Carbonation

**Figure 6:**
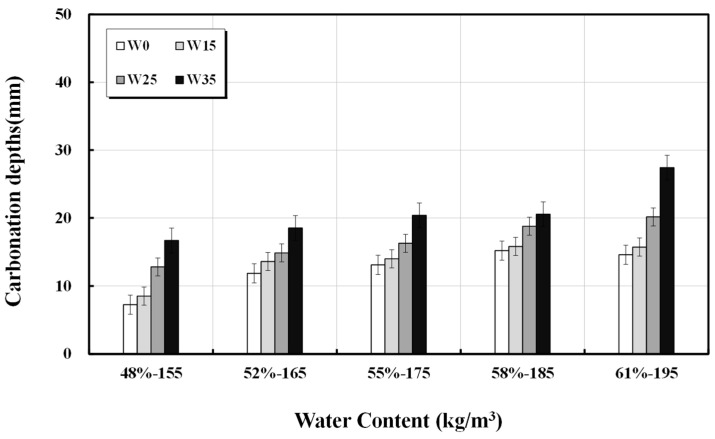
Depth of penetration of carbonation according to unit water content.

The results of accelerated carbonation for the 20 mixed designs with 15, 25, and 35 kg/m^3^ increases in unit water content for the five types of basic mixed designs are presented in Table 6, and the overall results are shown in Figure 6. When the additional unit water content was at least 25/m^3^, the concrete resistance to carbonation significantly decreased.

**Figure 7:**
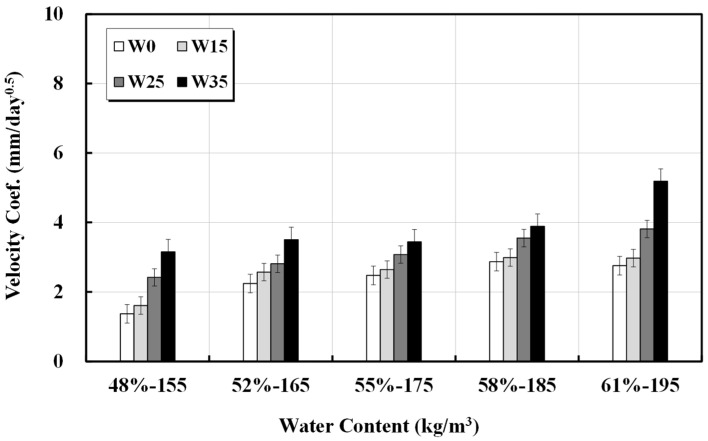
Velocity coefficient of carbonation against unit water content.

The calculation results of the carbonation rate coefficient are presented in Table 7 and Figure 7. The range of the carbonation rate coefficient of the five basic mixed designs was approximately 1.4–2.9 mm/day^0.5^, but it increased significantly to approximately 3.2–5.2 mm/day^0.5^ for mixes with an additional unit water content of 35 kg/m^3^. The increase in the carbonation rate coefficient was extremely high with the increase in unit water content for lower strength concretes having higher basic unit water content.

**Figure 8:**
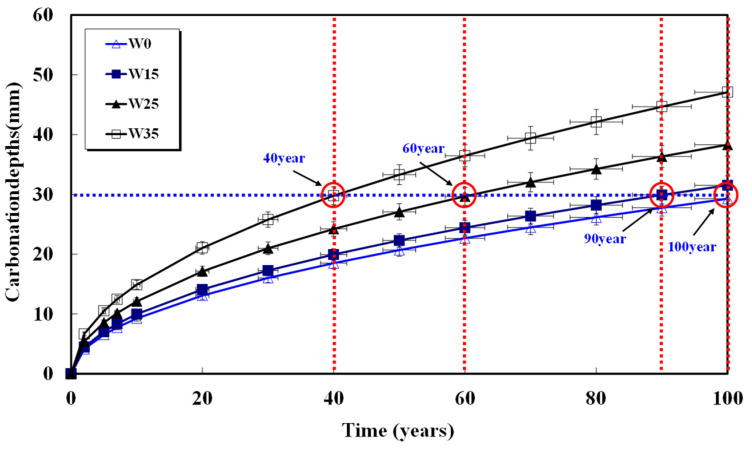
Carbonation depth of concrete with time (48%-155 kg).

**Figure 9:**
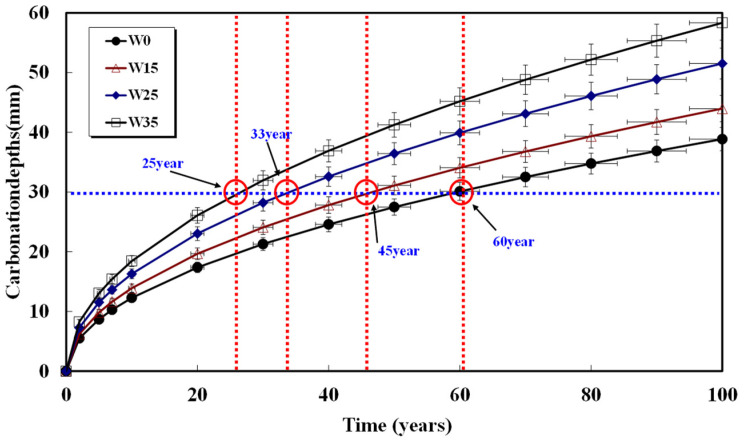
Carbonation depth of concrete with time (52%-165 kg).

**Figure 10:**
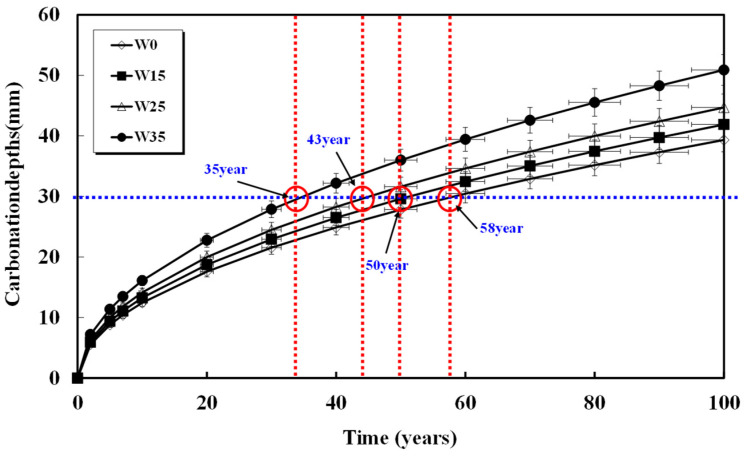
Carbonation depth of concrete with time (55%-175 kg).

**Figure 11:**
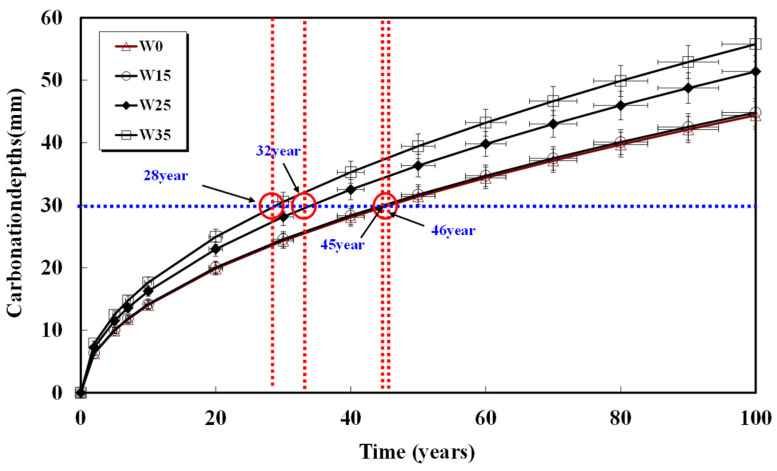
Carbonation depth of concrete with time (58%-185 kg).

**Figure 12:**
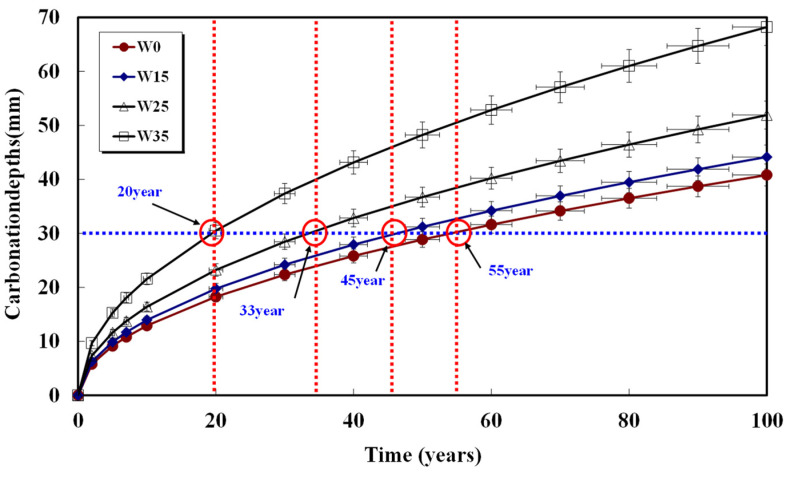
Carbonation depth of concrete with time (61%-195 kg).

Assuming a rebar cover thickness of 30 mm and calculating the required time for carbonation, the results are shown in Figure 8, Figure 9, Figure 10, Figure 11 and Figure 12 and presented in Table 8. When 15, 25, and 35 kg/m^3^ of water were added to each basic unit water content, the carbonation time was reduced by an average of 14%, 36%, and 52%, respectively. Notably, errors in unit water content or artificial control due to water addition during the concrete production process significantly reduced concrete compressive strength and the progression of long-term carbonation. Particularly in the concrete production process, even if the water content within the concrete inevitably increases as a result of quality control water additions, monitoring to keep the water increase at 15 kg/m^3^ or below is necessary to ensure durability against carbonation.

### 3.4. Porosity

**Figure 13:**
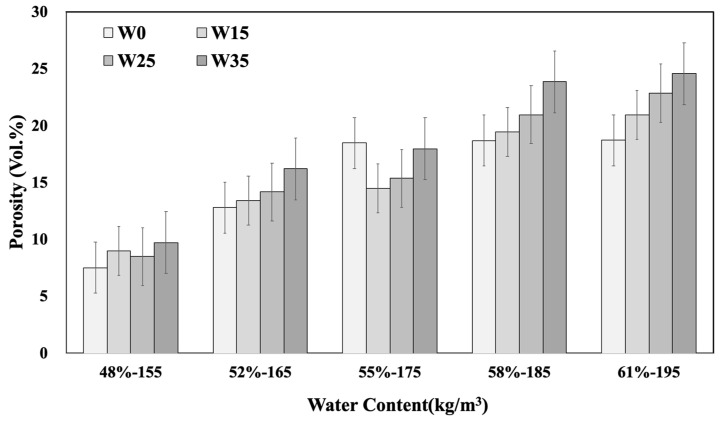
Depth of porosity according to unit water content.

This study evaluated concrete without AE agents. The evaluation of concrete porosity based on unit water content changes was compared using the porosity of the 20 mixed designs. The results are presented in Table 9 and Figure 13. A noticeable trend of increasing total porosity was observed as the unit water content increased. Especially for mixes with high W/C ratios, a significant increase in porosity, which is attributed to capillary pores formed by unreacted free water in the concrete mixed, was observed.

## 4. Discussion

**Figure 14:**
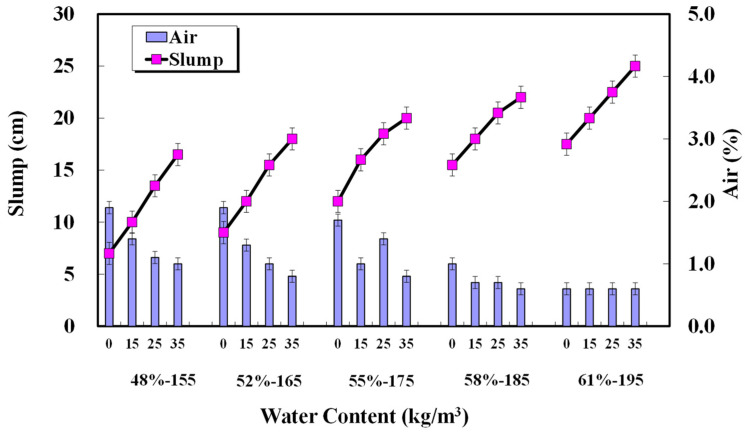
Changes in slump and air content according to unit water content.

The changes in slump and air content according to unit water content are presented in Figure 14. Changes in unit water content in concrete mixes without AE agents significantly affected concrete performance. An increase in a unit water content of 25 kg/m^3^ or higher may reduce the concrete compressive strength below the design standard strength and significantly affect the occurrence of bleeding water. An extra unit water content of at least 25 kg/m^3^ could significantly affect the diffusion coefficient of chloride ions within the concrete. Caution is required even in the production of normal-strength concrete, and for mixed designs requiring durability, a unit water content of 175 kg/m^3^ or less is considered effective. The impact of unit water content and water–cement ratio on concrete durability, especially regarding carbonation, was extremely high. This was similarly observed in the analysis of porosity. When 15, 25, and 35 kg/m^3^ of water were added to each basic unit water content, the carbonation time was reduced by an average of 14%, 36%, and 52%, respectively. In the ACI method, rounded coarse aggregates generally require 18 kg less water for non-air-entrained and 15 kg less for air-entrained concretes. If the measured unit quantity is larger than the unit quantity during the mix design process due to additional water, there is a risk of obtaining low-quality concrete. Lower strength, slump difference, and bleeding may occur than concrete design standards or required performance. Considering aggregate type, absorption rate, and moisture state, unit water content error management in concrete production processes requires greater attention. Further, many studies on water content control in concrete mixtures, maintaining the unit cement amount constant, have focused on aggregates, their absorption rates, and unit water content. When using recycled aggregate, the moisture state of the aggregate and unit water content significantly reduce concrete strength. If an aggregate with a high water absorption rate is used in a dry state, the aggregate absorbs water, resulting in slump loss and a decrease in the strength of the concrete. Conversely, wet aggregate supplies additional water to the concrete, which causes bleeding, material separation, and cracks. Based on the experimental scope and results of this study, a water–cement ratio of 48% or less and a unit water content of 155 kg/m^3^ or less are considered effective, and errors in unit water content occurring during the production process should be maintained at 15 kg/m^3^ or less.

## 5. Conclusions

This study showed that in the ready-mixed concrete production process, increases in concrete unit water content due to quality control errors and artificial water additions significantly reduced concrete compressive strength, increased porosity, and increased occurrence of bleeding. This also significantly reduced durability (resistance to chloride ion penetration and carbonation). When dividing concrete strength and purpose ranges from a durability perspective, changes in unit water content in the production of non-structural normal or low-strength concrete should be maintained at 25 kg/m^3^ or less. This includes preventing bleeding and concrete material separation. Meanwhile, in the production of concrete requiring at least normal strength or durability, the error or artificial change in unit water content should be maintained at 15 kg/m^3^ or less, and a water–cement ratio of 48% or less and a unit water content of 155 kg/m^3^ or less are considered effective in mixed design. Controlling the unit water content of concrete without AE agents to 15 kg/m^3^ or less is necessary to minimize performance degradation. Considering aggregate type, absorption rate, and moisture state, unit water content error management in concrete production processes requires greater attention. Future experimental studies on aggregate types (natural, crushed, and recycled aggregate) and their moisture states are required. Additionally, the effect of W/C ratio or unit water content may be different with types of supplementary cementitious materials, and further experimental studies are required.

## Figures and Tables

**Table 1:** Concrete mix proportion for specimens.

Specimens	W/C (%)	S/a (%)	Unit Weight (kg/m^3^)				
			Total Water Content		Cement	FA	CA
			Unit Water	Addition			
48%-155	48.4	45.7	155	0	320	885	1050
				+15			
				+25			
				+35			
52%-165	51.6	45.7	165	0	320	872	1036
				+15			
				+25			
				+35			
55%-175	54.7	45.7	175	0	320	860	1021
				+15			
				+25			
				+35			
58%-185	57.8	45.7	185	0	320	848	1007
				+15			
				+25			
				+35			
61%-195	60.9	45.7	195	0	320	836	993
				+15			
				+25			
				+35			

FA: fine aggregate; CA: coarse aggregate; S/a: fine aggregate to total aggregate rate.

**Table 2:** Physical properties of aggregates.

Category	Density (g/m^3^)	Fineness Modulus (%)	Absorption Rate (%)	Passing Rate through 0.08 mm Sieve (%)
Fine aggregate	2.64	2.57	1.42	3.00
Coarse aggregate	2.67	6.96	0.58	-

**Table 3:** Experimental items [25,26,27,28,29].

Experimental Items	Levels	Test Standards
Compressive Strength	Ages 3 d, 7 d, 28 d	KS F 2405
Chloride Ion Penetration Resistance	Age 28 d	NT BUILD 492
Accelerated Carbonation Resistance	Ages 7 d, 14 d, 21 d, 28 d	KS F 2584, KS F 2596
Porosity	Age 28 d	Mercury Intrusion Penetrometer

**Table 4:** Image analysis of chloride ion penetration.

	(Additional Water)	W0 (0 kg)	W15 (15 kg)	W25 (25 kg)	W35 (35 kg)
Specimen					
48%-155		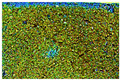	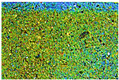	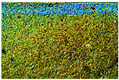	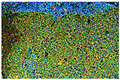
52%-165		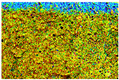	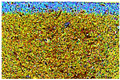	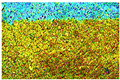	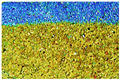
55%-175		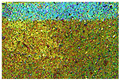	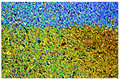	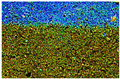	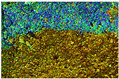
58%-185		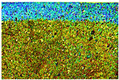	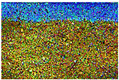	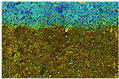	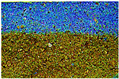
61%-195		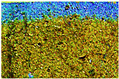	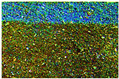	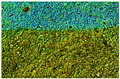	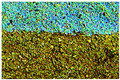

**Table 5:** Results of the chloride ion penetration resistance test.

Category			Chloride Ion Penetration Depth (m)	Chloride Ion Diffusion Coefficient (m^2^/s)
W/C-Unit Water-Add. Water (Specimen)		Additional Water (kg)		
48%-155	W0	0	7.50	6.28 × 10^−12^
	W15	15	8.73	7.48 × 10^−12^
	W25	25	7.58	6.35 × 10^−12^
	W35	35	13.56	1.21 × 10^−11^
52%-165	W0	0	9.59	8.29 × 10^−12^
	W15	15	9.95	8.62 × 10^−12^
	W25	25	9.51	8.22 × 10^−12^
	W35	35	12.82	1.14 × 10^−11^
55%175	W0	0	9.56	8.26 × 10^−12^
	W15	15	9.80	8.47 × 10^−12^
	W25	25	10.17	8.85 × 10^−12^
	W35	35	14.85	1.34 × 10^−11^
58%-185	W0	0	12.03	1.06 × 10^−11^
	W15	15	11.94	1.05 × 10^−11^
	W25	25	13.32	1.16 × 10^−11^
	W35	35	16.91	1.53 × 10^−11^
61%-195	W0	0	11.45	1.01 × 10^−11^
	W15	15	12.69	1.13 × 10^−11^
	W25	25	14.00	3.91 × 10^−11^
	W35	35	19.42	1.77 × 10^−11^

**Table 6:** Image analysis of concrete section by accelerated carbonation.

	48%-155	52%-165	55%-175	58%-185	61%-195
W0	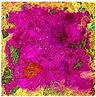	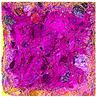	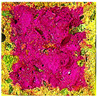	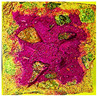	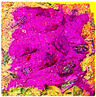
W15	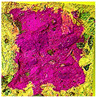	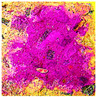	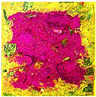	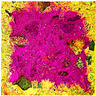	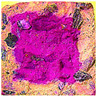
W25	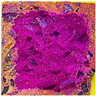	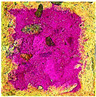	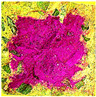	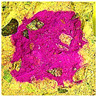	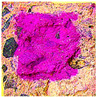
W35	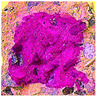	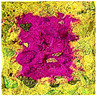	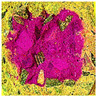	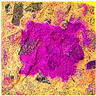	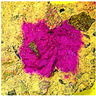

**Table 7:** Velocity coef. of carbonation according to unit water content.

Specimen	W0 (mm/day^0.5^)	W15 (mm/day^0.5^)	W25 (mm/day^0.5^)	W35 (mm/day^0.5^)
48%-155	1.37	1.61	2.42	3.15
52%-165	2.24	2.57	2.81	3.50
55%-175	2.48	2.64	3.07	3.44
58%-185	2.87	2.99	3.55	3.89
61%-195	2.76	2.97	3.81	5.18

**Table 8:** Carbonation progress time from concrete surface to 30 mm depth.

Specimen	W0 (Year)	W15 (Year)	W25 (Year)	W35 (Year)
48%-155	100	90	60	40
52%-165	60	45	33	25
55%-175	58	50	43	35
58%-185	46	45	32	28
61%-195	55	45	33	20

**Table 9:** Porosity characteristics according to unit water content.

Specimen	Porosity (vol. %)			
	W0	W15	W25	W35
48%-155	7.53	9.02	8.51	9.75
52%-165	12.81	13.42	14.19	16.24
55%-175	18.50	14.49	15.39	17.99
58%-185	18.71	19.45	20.99	23.87
61%-195	18.73	20.96	22.87	24.59

## Data Availability

Data are contained within the article.
